# Suicide trends in Denmark—An ecological study exploring suicide methods from 1995 to 2019

**DOI:** 10.1371/journal.pone.0296324

**Published:** 2023-12-29

**Authors:** Agnieszka Konieczna, Christina Petrea Larsen, Sarah Grube Jakobsen, Taro Okuda, Karin Moriyama, Winibaldus Stefanus Mere, Erik Christiansen

**Affiliations:** 1 Centre for Suicide Research, Odense, Denmark; 2 Research Unit of Health Promotion, Department of Public Health, University of Southern Denmark, Odense, Denmark; 3 Research Unit Mental Health, Children and Adult, Aabenraa, Department of Regional Health Research, University of Southern Denmark, Odense, Denmark; 4 Department of Regional Health Research, University of Southern Denmark, Odense, Denmark; 5 Faculty of Humanities and Director of Institute for Social Ethics, Nanzan University, Nagoya, Japan; 6 Faculty of Law and Institute for Social Ethics, Nanzan University, Nagoya, Japan; Universidade Federal do Rio Grande do Norte, BRAZIL

## Abstract

Suicide is a major public health problem and complex phenomenon, affecting many people around the world. However, the incidence of suicide varies by sex and age, which includes differences in the means used. Therefore, to implement effective preventative interventions, it is important to study these differences to design effective, preventative interventions. This study investigates the trends in suicide rates in Denmark from 1995 to 2019 by analysing changes based on sex, age, and the means used for suicide. Data on all suicide deaths in the study period were extracted from the Danish Register of Causes of Death, and data on the background population were obtained from Statistics Denmark. We used negative binomial regression models to analyse the data, and the obtained estimates as a logarithm of the rate ratios allowed us to compare the results across groups and years. An overall decline in Danish suicide rates was observed during the study period, with the exception of young females aged 15–29 years. The demographic composition did not change significantly, and suicide rates are still highest for males and the elderly aged 60+. Hanging, self-poisoning and firearms remain the most prevalent means of suicide. Suicide prevention initiatives are required, especially interventions targeting males and the elderly. Restricting access to the means of suicide for these groups with high fatality rates may help reduce the overall suicide rate. Moreover, more research is needed to understand the factors that lead to suicide and affect the choice of means, which should also include studying the effects of different suicide prevention strategies on males and females from different age groups.

## Introduction

Suicide is a serious public health problem that impacts the lives of many people. At least 700,000 people are estimated to die from suicide each year worldwide, and suicide accounts for 1.3% of all deaths [1]. The number of suicide deaths continues to increase globally, but the rate of suicide has dropped by 45.8% from 1990 to 2019, when accounting for population growth [1].

Suicide varies by age, sex, and means of suicide [2, 3]. Suicide rates are highest among males and the elderly, but suicide is also the second most frequent cause of death among young people [1, 4]. During the period from 1922 to 1991, Denmark struggled with high suicide rates. For both females and males, the suicide rate peaked in 1945 and 1980, respectively [5–7]. Since 1980, the rates have slowly declined to the present level (2019) at 12.49 (18.85 for males and 6.27 for females) per 100,000 person-years. An array of factors influences suicide risk, including biological, psychological, sociological, and demographic factors, but personal choice and access to the means of suicide are significant factors in fatal outcomes [2]. Previous studies have shown that for all groups, a high risk of suicide has been linked to two factors: gathering information on suicide and gaining access to means [8, 9].

The methods of suicide have fluctuated in Denmark from 1920 to 1991 [5–7]. Historically, hanging was the most prevalent method of suicide (70%) among males, until the period between 1945 to 1950, when a large percentage of suicides was caused by self-poisoning with household gas (67%) due to the easy access to coal gas [7]. In the 1920s, around half of the suicide deaths were by hanging. Since then, self-poisoning has been one of the most common methods for females [5–7]. However, self-poisoning accounted for a very large proportion of suicides among both males and females, with the rate increasing until 1950 [5]. After the Second World War, both females and males began to choose other suicide methods. These changes in methods occurred because poisonous coal gas was replaced by other, less dangerous gases [7]. Self-poisoning declined until the mid-1960s, until it increased again to a high level in the 1980s [5] because of over-the-counter analgesics, especially paracetamol [7]. At the end of this period, an increasing number of suicides were by hanging, drowning, shooting, and other methods [5–7]. Since then, suicide deaths have markedly declined.

Although the suicide rate has decreased since 1980, the number of suicides is still high in Denmark. Therefore, monitoring the development of suicide rates by sex, age and method is important, as this knowledge will help to empower and equip decision-makers to design and implement essential suicide prevention measures [10]. Our study focusses on examining any recent changes in the development of suicide methods and any correlation between a change in methods and a change of suicide rates by sex and age. These data might provide important information on whether changes in specific methods can clarify the variation in suicide rates. There have been no published Danish studies on the trends in suicide methods over the past 30 years; therefore, this study aims to address this knowledge gap.

Our study investigates the trends in suicide rates in Denmark during the period from 1995 to 2019 by analysing sex and age-specific changes in suicide rates and by examining the distribution of suicide methods among males and females categorized in different age groups.

## Materials and methods

Data on suicide deaths were extracted from the Danish Register of Causes of Death (COD) for the 25-year period of 1995 to 2019 [11]. Data on the background population were obtained from Statistics Denmark [12], and individuals aged < 15 years were excluded from this study due to the very low suicide rate.

The annual crude rates by sex and age were calculated as the number of suicides in a year, divided by the average of the population size. Suicide rates were expressed as the incidence rate (IR) of suicides per 100,000 person-years.

The individuals were divided into three age groups: 15–29 years (youth), 30–59 years (adult), and 60+ years (elderly). All suicide methods were categorised into eight groups according to the ICD-10 X-codes. These eight groups were then divided into two overall groups: “violent methods”, which included hanging (X70), firearms (X72–X75), sharp objects (X78), jumping from heights (X80), and jumping in front of or crashing with moving objects (X81-X82); and “non-violent and other methods”, which included self-poisoning (X60– X69), drowning (X71) and other methods [13, 14]. “Other methods” included methods accounting for less than 2% of the overall number of suicides: fire (X76), hot vapours (X77), cutting/piercing with a blunt object (X79), other specified and classifiable means (X83), other unspecified means (X84), and the sequelae of intentional self-harm (Y87.0) [15].

### Statistical analysis

Changes in the overall suicide rates from 1995 to 2019 were analysed by a negative binomial regression using the suicide rate as an outcome variable and the time variable year as a linear term. Negative binomial regression models were used because of Poisson overdispersion in the data. Time trends by sex (and age) were tested by including the interaction term between year and sex (and age) in the model. A t-test and chi-square independence test were used to examine differences in age means and the distribution of methods between males and females.

We calculated the absolute number and the percentage distribution of suicide methods by age and sex. We divided the study period 1995 to 2019 into five-year time periods and calculated the suicide rates by methods for each period. The incidence rates were calculated by summing up the total amount of incidences divided by the total amount of time in years (person years).

To assess time trends in suicide methods by sex and for each age group by sex, we used negative binomial regression models with method-specific suicide rates as outcome variables and year as a linear term. Each model included sex or age group and an interaction term between year and sex or age group. We examined trends in all the methods categories (i.e., violent and non-violent and other methods) and then estimated trends for each method of suicide.

Negative binomial regression models were conducted using the procedure PROC GENMOD on SAS, version 9.4. Negative binomial regression coefficients were used as the measure of trends along with 95% confidence intervals and p-values (significance level of 5%). To determine significant changes in the rates by sex and age, p-values for interaction were calculated.

## Results

### Changes in suicide rates by sex

A total of 16,705 suicides were reported in Denmark during the 25-year study period. Of these suicides, 12,058 were males (72%) and 4,647 females (28%). The mean age was 53.8 (SD = 18.4, range = 15–103), and males were significantly younger than females at the time of death (p<0.0001), but the mean age difference was only 3.4 and the SDs were the same (males: 52.9 years (SD = 18.4, range = 15–103); females: 56.3 years (SD = 18.1, range = 15–101). The annual suicide rate in Denmark decreased by 41.5% from 21.35 per 100,000 in 1995 to 12.49 per 100,000 in 2019. The overall decrease in the rate for this period was higher for females, 53.7% (13.55 to 6.27 per 100,000), than for males, 36.1% (29.48 to 18.85 per 100,000), p<0.0001.

The negative binomial regression model showed a significant decrease in the suicide rate by 2.1% per year (p < .0001). The suicide rate for males decreased significantly by 1.9% per year (p < .0001) and by 2.6% for females per year (p < .0001). However, the change in suicide rates differed significantly between males and females (p<0.0370).

The negative binomial regression model showed a significant decrease in the suicide rate by 2.1% per year (95% CI: [-2.4,-1.7], p < .0001). The suicide rate for males decreased significantly by 1.9% per year (95% CI: [-2.3, -1.6], p < .0001) and by 2.6% for females per year (95% CI: [-3.1, -2.0], p < .0001). However, the change in suicide rates differed significantly between males and females p<0.0370).

### Changes in suicide rates by age

**Fig 1:**
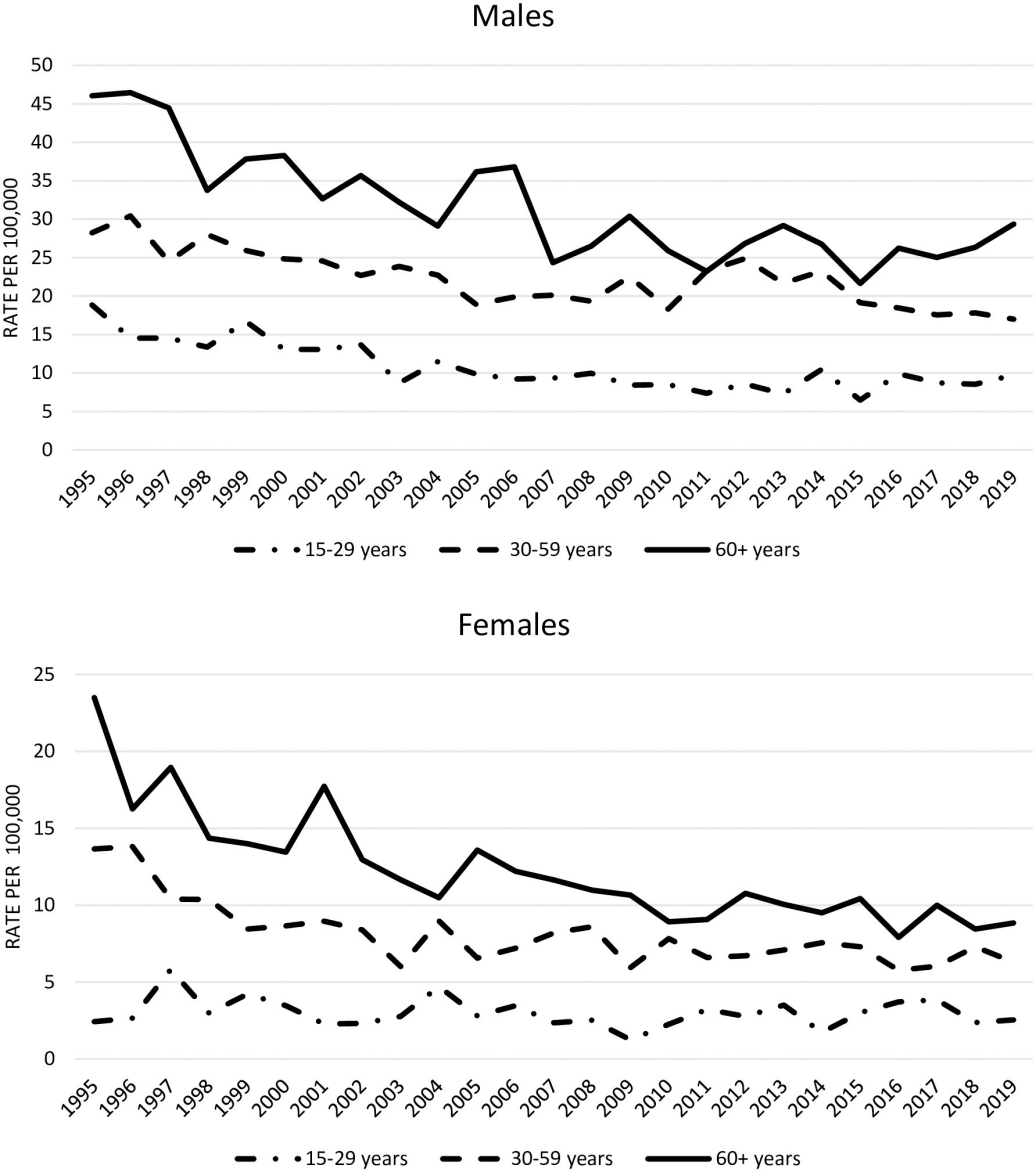
Time-trends of suicide rates for males and females by age in Denmark, 1995 to 2019.

Fig 1 shows trends in the incidence rates of suicide by age group. In males, the rates declined in all age groups: from 18.84 to 9.73 by 48.4% in the age group 15–29 years; from 28.23 to 17.00 by 39.8% in the age group 30–59 years, and from 46.07 to 29.39 by 36.2% in the age group 60+ years. For females, the rate in the age group 15–29 years plateaued with a small increase from 2.41 in 1995 to 2.54 in 2019. The suicide rate declined in the age group 30–59 years by 54% from 13.66 to 6.29, and by 62.4% in the age group 60+ from 23.49 to 8.84.

The negative binomial regression model showed a significant decrease by 3.0% per year in males aged 15–19 years (95% CI: [-3.8,-2.2], p < .0001). For females aged 15–19 years, there was a small, non-significant decrease of 0.9% per year (95% CI: [-2.5, 0.7], p < .274). Among males aged 30–59 years, the suicide rate decreased significantly by 1.7% per year (95% CI: [-2.2,-1.2], p < .0001), and females in this age group had an even greater decrease by 2.5% per year (95% CI: [-3.3,-1.8], p < .0001). Suicide rates among males aged 60+ significantly decreased by 2.4% per year (95% CI: [-3.0,-1.7), whereas a significant decrease by 3.3% per year was seen for females in the same age group (95% CI: [-4.0,-2.6], p < .0001). Trend differences between age groups were significant in males, and the largest decline was seen in the youngest age group. The differences between age groups were also significant in females (p = 0.017), and the largest decline was seen in the oldest age group. No trend differences were seen between age groups for the overall rate.

### Methods of suicide

Of all suicides, 10,609 (63.5%) (males: n = 8,510, 70.6% and females: n = 2,099, 45.2%) died by “violent methods” and 6,096 (36.5%) (males: n = 3,548, 29.4% and females: 2,548, 54.8%) died by “non-violent and other methods”. Hanging was the most usual method (39.57%), followed by self-poisoning (28.52%), and firearms (10.16%). Males chose suicide by hanging and firearms more often than females (43.16% vs. 30.26% and 13.74% vs. 0.86%, respectively), whereas self-poisoning and drowning were more frequently used by females (23.15% vs. 42.46% and 4.53% vs 10.65%, respectively). The test of independence indicated that the distribution of methods differed between males and females (p<0.0001).

The distribution of methods by sex and age are shown in Table 1. Hanging was the most preferred method for males in all age groups, followed by self-poisoning and firearms. Among females, those aged 15–29 years used violent methods most frequently, whereas non-violent and other methods were most frequent in the other age groups. Hanging and self-poisoning were the most dominant methods for young females, whereas self-poisoning followed by hanging and drowning were common in the other female age groups.

**Table 1:** Absolute number and percentage of suicides by age, sex and suicide methods, total share for 1995 to 2019.

	15–29 years		30–59 years		60+ years	
	N = 1,788 (%)		N = 8,659 (%)		N = 6,258 (%)	
**Suicide methods**						
**Male**						
*All violent*	1,088	(77.1)	4,351	(68.1)	3,071	(72.1)
*All non-violent and other*	323	(22.9)	2,039	(31.9)	1,186	(27.9)
Self-poisoning	254	(18.0)	1,68	(26.3)	857	(20.1)
Hanging	660	(46.8)	2,679	(41.9)	1,865	(43.8)
Drowning	44	(3.1)	231	(3.6)	271	(6.4)
Firearms	171	(12.1)	801	(12.5)	685	(16.1)
Sharp objects	24	(1.7)	268	(4.2)	212	(5.0)
Jumping	87	(6.2)	279	(4.4)	219	(5.1)
Moving objects	146	(10.4)	324	(5.1)	90	(2.1)
Other methods	25	(1.8)	128	(2.0)	58	(1.4)
**Female**						
*All violent*	261	(69.2)	1,003	(44.2)	835	(41.7)
*All non-violent and other*	116	(30.8)	1,266	(55.8)	1,166	(58.3)
Self-poisoning	102	(27.1)	1,021	(45.0)	850	(20.0)
Hanging	179	(47.5)	632	(27.9)	595	(14.0)
Drowning	10	(2.7)	205	(9.0)	280	(6.6)
Firearms	7	(1.9)	27	(1.2)	6	(0.1)
Sharp objects	4	(1.1)	88	(3.9)	68	(1.6)
Jumping	27	(7.2)	159	(7.0)	130	(3.1)
Moving objects	44	(11.7)	97	(4.3)	36	(0.8)
Other methods	4	(1.1)	40	(1.8)	36	(0.9)

### Trends in suicide rates by methods

During 1995 to 2019, there was a significant decrease in violent suicides by 1.8% per year (95% CI: [-2.1,-1.4], p < .0001) and a significant decrease in non-violent and other suicides by 2.6% per year (95% CI: [-3.2,-2.0], p < .0001).

Table 2 shows trends in suicide methods by sex. Males used violent methods more often than females (15.34 vs 3.66 per 100,000), and this change in trend was significantly stronger for females than males. The non-violent and other suicide rate was higher in males (6.39 per 100,000) than in females (4.44 per 100,000), but this change in trend was not significantly different for the two sexes. Moreover, the changes in suicide by hanging and jumping from heights were significantly different between males and females, with a markedly stronger decline for females than males (not shown).

**Table 2:** Changes in suicide methods over time in Denmark by sex, 1995–2019.

Method of suicide	Sex	Percent change per year	95% CI	P-value	Sex differences
**All violent**	Males	-1.6	-2.0 to -1.3	**<0.0001**	**p = 0.009**
	Females	-2.6	-3.6 to -2.0	**<0.0001**	
**All non-violent and other**	Males	-2.8	-3.7 to -1.9	**<0.0001**	p = 0.950
	Females	-2.7	-3.5 to -2.0	**<0.0001**	

Abbreviations: CI: confidence interval

Table 3 shows trends in the incidence rate of suicide methods by age group and sex over time. A significant decrease was seen in the rates of suicides by self-poisoning, hanging, drowning, and firearms in males of all ages. The use of self-poisoning and firearms had decreased more rapidly in the age group 15–29 years than in the older age groups. All males had an insignificant upward trend in suicide by sharp objects. Likewise, the increase in suicide by other methods was not statistically significant among males in the age groups 15–29 years and 30–59 years. A significant decrease in jumping and an increase in other methods was seen in the age group 60+ years. In all male age groups no significant changes were seen for suicide by moving objects, nor in suicide by jumping in the age groups 15–29 years and 30–59 years. The greatest reduction in the suicide rate among males was evident for self-poisoning and firearms for the 15–29 years age group and for suicide by other methods for the age group 60+ years.

**Table 3:** Changes in suicide methods by sex and age group over the period 1995 to 2019 (baseline: 1995–1999).

	Incidence rates per 100,000 person-years					Negative Binomial regression		
**Males**								
**Method of suicide**	**1995–1999**	**2000–2004**	**2005–2009**	**2010–2014**	**2015–2019**	**Percent change by year**	**95% CI**	**P-value**
**Age 15–19**								
*All Violent*	11.17	9.16	7.66	6.85	6.92	-2.5	-3,3 to -1,7	***
*All Non-violent and other*	4.47	2.85	1.69	1.60	1.76	-4.7	-6,7 to -2,7	***
Self-poisoning	3.65	2.32	1.19	1.41	1.16	-5.6	-7,6 to -3,6	***
Hanging	6.33	5.09	4.90	4.19	4.78	-1.5	-2,6 to -0,4	*
Drowning	0.67	0.37	0.21	0.11	0.32	-4.9	-9,2 to -0,4	*
Firearms	2.53	1.79	1.19	0.69	0.42	-8.4	-10,4 to -6,	***
Sharp object	0.19	0.12	0.12	0.19	0.28	3.2	-2,5 to 9,1	
Jumping	0.78	0.81	0.62	0.72	0.42	-2.1	-4,9 to 0,7	
Moving objects	1.34	1.34	0.82	1.07	1.02	-1.7	-3,8 to 0,5	
Other methods	0.15	0.16	0.29	0.08	0.28	2.5	-3,5 to 8,9	
**Age 30–59**								
*All Violent*	17.07	17.20	13.74	14.84	13.08	-1.3	-1,8 to -0,8	***
*All Non-violent and other*	10.33	6.54	6.40	7.43	4.91	-2.7	-3,7 to -1,6	***
Self-poisoning	8.55	5.28	5.18	6.31	4.03	-2.7	-3,8 to -1,5	***
Hanging	10.05	10.77	8.53	9.02	8.37	-1.0	-1,7 to -0,3	*
Drowning	1.37	0.90	0.69	0.58	0.48	-4.8	-6.8 to -2.8	***
Firearms	3.65	3.46	2.42	2.72	1.70	-3.2	-4.3 to -2.1	***
Sharp object	0.95	0.80	0.85	1.01	1.08	0.7	-1.2 to 2.5	
Jumping	1.35	0.85	0.83	0.87	0.98	-1.4	-3.1 to 0.2	
Moving objects	1.07	1.31	1.11	1.22	0.94	-0.6	-2.1 to 0.9	
Other methods	0.40	0.36	0.54	0.53	0.41	1.3	-1.2 to 3.8	
**Age 60+**								
*All Violent*	29.38	24.14	22.83	19.09	18.49	-2.3	-3.0 to -1.6	***
*All Non-violent and other*	12.30	9.36	7.88	7.32	7.29	-2.6	-3,3 to -1,7	***
Self-poisoning	9.50	6.89	5.00	5.40	5.24	-2.9	-4.0 to -1,9	***
Hanging	17.96	16.00	13.53	11.74	10.34	-2.7	-3,5 to -2.0	***
Drowning	2.63	2.35	2.30	1.50	1.39	-3.1	-4,7 to -1,5	**
Firearms	6.46	4.74	5.54	4.29	4.24	-2.0	-3,1 to -0,8	**
Sharp object	1.69	1.39	1.75	1.07	1.78	0	-2.0 to 2.0	
Jumping	2.45	1.55	1.46	1.30	1.39	-2.9	-4,7 to -1.0	*
Moving objects	0.80	0.46	0.55	0.68	0.74	0.2	-2,7 to 3,1	
Other methods	0.18	0.13	0.58	0.42	0.65	7.3	2,1 to 12,9	*
**Females**								
**Method of suicide**	**1995–1999**	**2000–2004**	**2005–2009**	**2010–2014**	**2015–2019**	**Percent change by year**	**95% CI**	**P-value**
**Age 15–19**								
*All Violent*	2.05	2.21	1.62	1.94	2.50	0.6	-1,3 to 2,5	
*All Non-violent and other*	1.55	0.88	0.85	0.75	0.59	-4.2	-6,7 to -1,8	**
Self-poisoning	1.31	0.75	0.77	0.67	0.55	-3.7	-6,4 to -0,9	*
Hanging	1.28	1.55	0.94	1.42	1.87	1.5	-0,6 to 3,5	
Drowning	0.15	0.13	0.04	0.08	0.00	-9.8	-18,2 to -0,5	*
Firearms	0.08	0.08	0.09	0.04	0.00	-7.0	-16,6 to 3,7	
Sharp object	0.08	0.00	0.00	0.04	0.04	-3.0	-15,3 to 11	
Jumping	0.23	0.25	0.30	0.12	0.18	-1.9	-6,8 to 3,4	
Moving objects	0.39	0.33	0.30	0.32	0.40	0.2	-3,7 to 4,4	
Other methods	0.08	0.00	0.04	0.00	0.04	-5.4	-17,6 to 8,8	
**Age 30–59**								
*All Violent*	4.82	3.57	3.10	3.15	3.26	-1.9	-3.0 to -0,9	**
*All Non-violent and other*	6.48	4.62	4.19	4.01	3.28	-3.1	-4,2 to -2,2	**
Self-poisoning	4.94	3.60	3.40	3.47	2.79	-2.5	-3,5 to -1,5	***
Hanging	2.95	2.24	1.94	2.02	2.12	-1.5	-2,8 to -0,3	*
Drowning	1.43	0.89	0.67	0.36	0.31	-7.6	-9,5 to -5,6	***
Firearms	0.09	0.12	0.11	0.05	0.11	0.4	-5,1 to 6,2	
Sharp object	0.43	0.38	0.28	0.20	0.27	-3.2	-6.0 to -0,3	*
Jumping	0.87	0.58	0.48	0.47	0.45	-3.9	-6,3 to -1,6	*
Moving objects	0.47	0.24	0.30	0.41	0.31	-1.2	-4,1 to 1,7	
Other methods	0.11	0.12	0.12	0.18	0.18	3.4	-1.0 to 8.0	
**Age 60+**								
*All Violent*	7.98	5.69	5.03	4.11	2.96	-4.6	-5,5 to -3,6	***
*All Non-violent and other*	9.43	7.54	6.75	5.56	6.15	-2.4	-3,3 to -1,6	**
Self-poisoning	6.56	5.09	5.24	4.28	4.57	-1.9	-2,9 to -1.0	***
Hanging	6.12	4.33	3.51	2.57	1.95	-5.4	-6,6 to -4,3	***
Drowning	2.64	2.08	1.33	1.23	1.32	-4.0	-5,9 to -2.0	***
Firearms	0.03	0.10	0.00	0.03	0.03	-3.3	-14,1 to 8,9	
Sharp object	0.37	0.26	0.39	0.45	0.52	2.0	-1,6 to 5,7	
Jumping	1.12	0.89	0.94	0.78	0.29	-5.0	-7,5 to -2,6	**
Moving objects	0.34	0.10	0.18	0.28	0.18	-1.0	-5,4 to 3,6	
Other methods	0.24	0.36	0.18	0.06	0.26	-2.0	-6,9 to 3.0	

*p<0.05.

**p<0.001.

***p<0.0001;

Abbreviations CI: confidence interval

Significant declines for suicide by self-poisoning and drowning were seen among females in all age groups. Younger females showed an upward trend in violent methods, but this increase was non-significant. Additionally, a non-significant, small increase was seen in this group for suicide by hanging and by moving objects. Moreover, a significant downward trend for suicide by hanging, drowning, sharp objects and jumping was seen for females aged 30–59 years, but an non-significant upward trend in suicides by other methods was also seen for this group. A significant decrease in suicide by hanging and jumping and non-significant increase in suicide by sharp objects was seen for the age group 60+ years. No significant changes were observed for firearms, sharp objects, jumping, and other methods in the youngest group, for firearms and moving objects in the middle-aged group, and for firearms, moving objects and other methods in the oldest age group. The greatest reductions in suicide rates among females were seen for drowning and firearms in the youngest age group, drowning in the middle-aged group, and hanging in the oldest age group.

Significant age differences were seen in all violent methods for both males and females, and significant differences were not seen in all non-violent and other methods. For males, a significant interaction of age with time was observed for hanging and firearms, and for females, with hanging, drowning, and jumping. In all cases, a reduction was observed except for hanging among females aged 15–29 years (Table 3 and S1 Fig).

## Discussion

This study investigated time trends in suicide rates in Denmark during the period from 1995 to 2019. We found a significant reduction in the overall suicide rate, with an average, annual reduction of around 2% for both sexes. For males, the greatest reduction was seen in the youngest age group. For females, the greatest reduction was seen in the oldest age group. Among all groups except youngest group of females, reductions were also seen for all violent methods, as well as all non-violent and other methods.

An earlier Danish study reported that suicide rates have declined in Denmark since 1980 [16]. This study adds new information on changes in suicide methods. Our finding of a decline in suicide rates could be explained by several factors. First, Denmark has experienced significant changes in both the access to healthcare and in the access to suicide means [17, 18]. Second, more effective medical treatment has been developed, including better treatment of mental illness and increased suicide prevention psychoeducation. During the last 20 years, suicide treatment units have opened in every region of the country, and treatment of suicidal behaviour has become a specialised effort in many psychiatric departments [18].

The suicide rates are significantly higher for males than for females, but the decline in suicide rates was seen in both sexes during the study period. A possible explanation for our finding can be that the initiated prevention strategies are likely to have benefitted both sexes, although not equally for all age groups. If prevention strategies in general have any effect on suicide rates, this study found a lack of effect on young females [19]. In the youngest group of females, violent methods had not been reduced, especially hanging. We do not know if this finding is related to the increasing prevalence of depression and anxiety among girls and young females [20], social/personal challenges in life, or the high incidence of self-harm and suicide attempts in this age group [21]. A study by Ruch et al. (2019) revealed that hanging and suffocation have become increasingly common ways of dying by suicide among girls and young females [22]. Another commonly used method is self-poisoning [22]. Interpreting the data is challenging because of the low number of suicides in the youngest group of females.

Males tend to choose violent methods, such as hanging and firearms, whereas females often choose poisoning or drowning. Poisoning with pesticides is common in many Asian countries and in Latin America [1], whereas suicide by firearms is the most common method in the US [23]. Our findings on the choice of suicide method corresponds with previous findings on common suicide methods in Europe [15].

We found large reductions for almost every method in the youngest group of males, except for hanging, which accounts for most suicides. The number of suicides by self-poisoning and firearms have significantly reduced, which could be explained by limited access to these means or a change in preferences. Some drugs (painkillers) are difficult to buy in Denmark, and firearms are restricted due to gun control legislation in Denmark. Moreover, many over-the-counter drugs have become less lethal when overdosed. The changes in self-poisoning could partly be explained by the introduction of restricting the package size of drugs [24]. Hanging was the most frequently used method in all male age groups, and this number had not been reduced in contrast to the category of other violent methods. Preventing suicide by hanging is difficult because the access to this means is easy, and this method is lethal [25].

Females are more likely to use non-violent methods, which are less lethal and easier to prevent. For the 60+ females, a large, overall reduction was seen for all violent methods. The significant decline in the suicide rate among females in the 60+ age group might be explained by effective suicide prevention initiatives and the fewer deaths from hanging and jumping [19]. The increase of incidence rates by the ’other methods’ category over the reported period may be explained by the emergence of a new method. The data in this category showed an increase of suicide by carbon monoxide poisoning by using charcoal grills indoors. Moreover, the internet and social media may contribute to the spread of new methods, as well as access to suicide prevention resources; thus, influencing the suicide rates.

### Implications for suicide prevention

The overall downward trend of the suicide rate appears to have plateaued in the last decade. However, the lack of a significant decline in suicides among females aged 15–29 years over the past 25 years is of concern. The trend of using more violent methods, especially hanging, indicates that young females might be turning towards more lethal means. At the same time, the suicide rate remains high in males and in people aged 60+. These findings indicate that further studies on suicide methods over time are needed to understand these trends. Males are far less likely than females to talk about their problems and to seek treatment for issues such as depression, substance abuse, and stressful life events, which makes suicide prevention more challenging [26–29].

Little is known about the various factors that contribute to the choice of a suicide method. Some studies have suggested sociocultural acceptability [30] and media portrayals of suicide [31]. A recent study by Marzano et al. [32] revealed that the choice of method depends on how lethal, easy, quick, accessible and/or painless the method is. Impulsivity and the presence of (and the impact from) other people, especially family, and fears of injury and survival also influence the choice of hanging: a study from the UK reported that hanging is considered to be a quick, painless, and clean method [30]. Moreover, materials for hanging are easily accessible, and this method does not require specific planning or technical knowledge [33]. A study in Switzerland confirmed that hanging is often carried out when there is only little suspicion of suicide [25]. In fact, suicide by hanging is the most used method, and it is also the most difficult suicide method to prevent. The wide availability of ligature points and ligatures makes it impossible to prevent this method. However, recommendations to reduce the risk of suicide by hanging include identifying those who might choose this method, while reducing or removing any potential ligature points. Additional recommendations include developing awareness campaigns about the catastrophic consequences of a failed hanging attempt and failed suicide attempts, in general [34–36].

Internationally, there are big differences in both suicide rates and suicide methods. However, when one suicide method becomes more difficult to access, it does not generally lead to another method being used instead [34, 35]. Other studies in Canada on the other hand demonstrated a switch from firearms to hanging after implementation of gun controls [36]. This calls for a focus on regulation and monitoring of other suicide methods, if possible in order to reduce the overall suicide rate. International knowledge about suicide, suicide methods, and suicide prevention initiatives are essential and inspire further research to reduce and prevent suicidal behaviour. Denmark is a country that has a highly developed health care system [18] coupled with a tradition of a valid and detailed registry of causes of death. Together, these national sources of data make it possible to identify changes in the trends of suicidal behaviour, knowledge that is also relevant for other countries since any changes in methods and trends can be further studied by using the data from the present suicide rates in Denmark.

### Strengths and limitations

Denmark’s national data coverage is an advantage of our data analysis, and the large study population increases the statistical strength of the estimates in this study. The quality of the suicide data depends on the physician’s notification and correct registration in the COD register [17]. In Denmark, the completeness of the COD register is considered high (≥ 90%) [37]. Changes in registration practice during the period from 1995 to 2019 and some level of underreporting might have influenced the suicide numbers. Nevertheless, studies by Tøllefsen et al. showed that the decreasing suicide rate could not be explained by misclassifications alone [38, 39]. In the ICD-10, deaths classified as "undetermined intent" are a separate category, and our study only included those cases categorized as "certain" suicides, which increases its external validity.

Another limitation is that this study cannot document a causal relationship between preventive efforts and the declining suicide rate, which is often difficult for observational studies, in general. Lastly, the data in this paper are by nature ecological and therefore based on aggregate measures, which means that this study can only include a limited number of explanatory variables. In addition, factors that may have influenced the trends were not included in these analyses.

## Conclusion

In general, suicide rates by all methods have declined, and some methods more than others. Although, substitution of suicide method may occur, the data and the analyses in this study cannot determine that. If suicide prevention strategies have any effect on suicide rates, we did not find that effect on young females since suicide rates for violent methods did not decline during the study period. The end of the study period was characterized by a non-declining trend in the suicide rates for females. This finding is important for decision makers to reduce the suicide rate. Our study shows that suicide by hanging has been reduced to the lowest rate of incidence for all groups across age and sex and yet hanging might still be the most challenging method to prevent. The differences in suicide and suicide method reduction in this study show that different suicide prevention strategies are needed for males and females at different ages. Denmark has a wide range of suicide prevention strategies employed at schools, in communities, and within primary care settings. However, there is still insufficient knowledge about which types of suicide prevention interventions are most effective in changing suicide behaviours, including access to methods, for both sexes of all ages. In addition, more research is needed to investigate the potential for substitution of suicide method.

## Supporting information

S1 Fig Suicide methods rate by age and sex. (TIF)
