# Supplementary figures and images for: Suicide trends in Denmark—An ecological study exploring suicide methods from 1995 to 2019
Source: PLoS One. 2023 Dec 29;18(12):e0296324. doi: 10.1371/journal.pone.0296324 (PMC10756527; doi:10.1371/journal.pone.0296324)

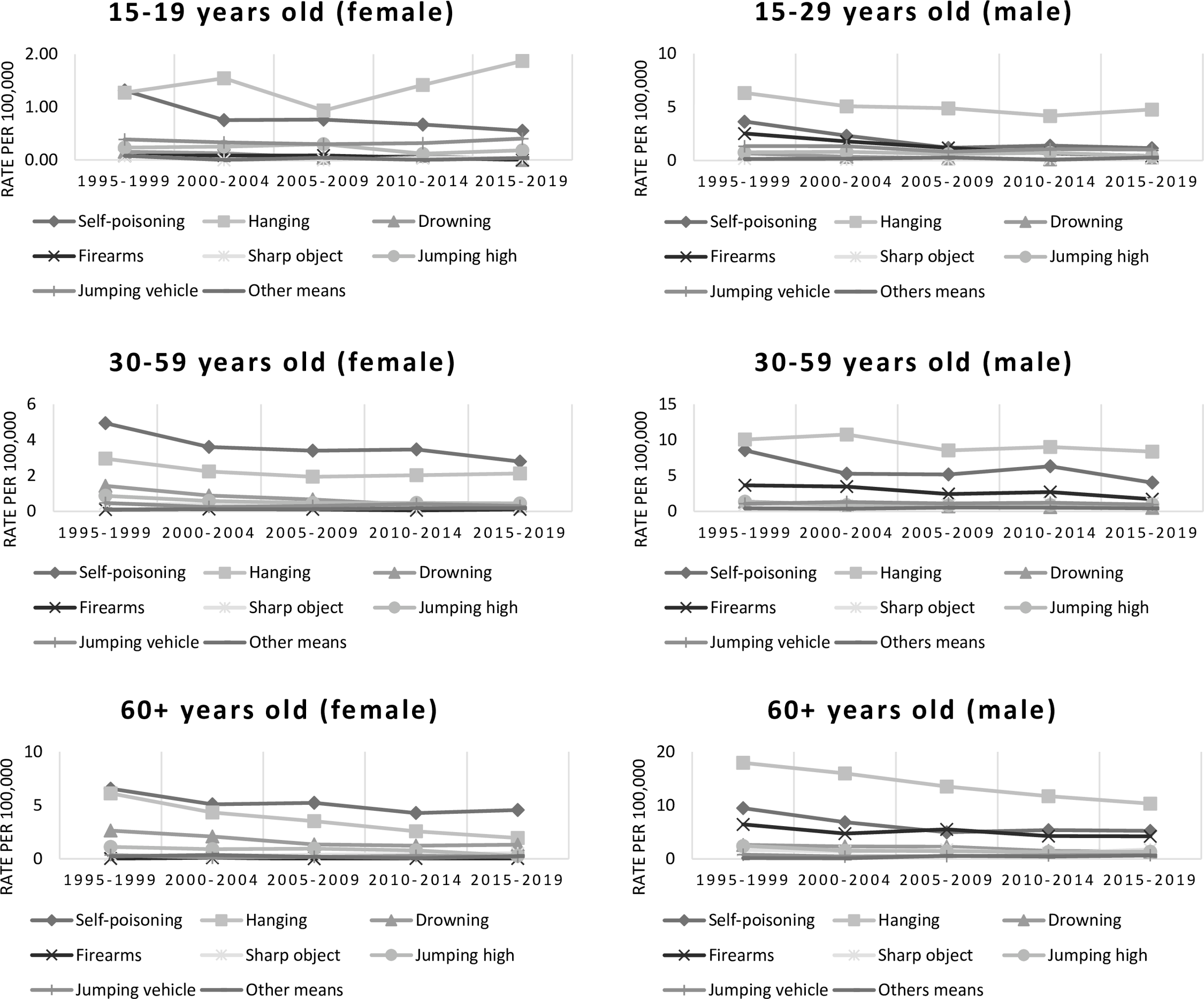

Supplement: S1 Fig — (TIF) [file pone.0296324.s001.tif]
